# Comparative profiling of surgically resected primary tumors and their lymph node metastases in small-cell lung cancer

**DOI:** 10.1016/j.esmoop.2025.104514

**Published:** 2025-03-18

**Authors:** K. Csende, B. Ferencz, K. Boettiger, M.D. Pozonec, A. Lantos, A. Ferenczy, O. Pipek, A. Solta, B. Ernhofer, V. Laszlo, E. Megyesfalvi, K. Schelch, V. Pozonec, J. Skarda, V. Skopelidou, Z. Lohinai, C. Lang, L. Horvath, K. Dezso, J. Fillinger, F. Renyi-Vamos, C. Aigner, B. Dome, Z. Megyesfalvi

**Affiliations:** 1 Department of Thoracic Surgery, Semmelweis University and National Institute of Oncology, Budapest, Hungary; 2 National Koranyi Institute of Pulmonology, Budapest, Hungary; 3 Department of Thoracic Surgery, Comprehensive Cancer Center, Medical University of Vienna, Vienna, Austria; 4 Department of Obstetrics and Gynecology, South Buda Central Hospital, Saint Emeric University Teaching Hospital, Budapest, Hungary; 5 Department of Physics of Complex Systems, Eotvos Lorand University, Budapest, Hungary; 6 Department of Thoracic and Abdominal Tumors and Clinical Pharmacology, National Institute of Oncology, Budapest, Hungary; 7 Multidisciplinary Centre of Head and Neck Tumors, National Institute of Oncology, Budapest, Hungary; 8 Institute of Clinical and Molecular Pathology, Medical Faculty, Palacký University Olomouc, Olomouc, Czech Republic; 9 Department of Pathology, University Hospital Ostrava, Ostrava, Czech Republic; 10 Faculty of Medicine, University of Ostrava, Ostrava, Czech Republic; 11 Torokbalint County Institute of Pulmonology, Torokbalint, Hungary; 12 Division of Pulmonology, Department of Medicine II, Medical University of Vienna, Vienna, Austria; 13 Department of Pathology and Experimental Cancer Research, Budapest, Hungary; 14 National Institute of Oncology and National Tumor Biology Laboratory, Budapest, Hungary; 15 Department of Translational Medicine, Lund University, Lund, Sweden

**Keywords:** small-cell lung cancer, subtypes, intertumoral heterogeneity, metastasis

## Abstract

**Background:**

Profiling studies in small-cell lung cancer (SCLC) have mainly focused on primary tumors, omitting the potential molecular changes that might occur during lymphatic metastasis formation. Here, we assessed the molecular discordance between primary SCLCs and corresponding lymph node (LN) metastases in the light of subtype distribution and expression of clinically relevant proteins.

**Methods:**

Comparative profiling of 32 surgically resected primary SCLCs and their LN metastases was achieved by RNA expression analysis and immunohistochemistry (IHC). In addition to subtype markers (ASCL1, NEUROD1, POU2F3, and YAP1), the expression of nine cancer-specific proteins was evaluated.

**Results:**

The selected clinically relevant molecules showed no significant differences in their RNA expression profile when assessing the primary tumors and their corresponding LN metastases. Nevertheless, IHC analyses revealed significantly higher DLL3 expression in the primary tumors than in the LN metastases (*P* = 0.008). In contrast, NEUROD1 expression was significantly lower in the primary tumors (versus LN metastases, *P* < 0.001). No statistically significant difference was found by IHC analysis in the case of other clinically relevant proteins. Concerning SCLC molecular subtypes, a change in subtype distribution was detected in 21 cases. Phenotype switching from neuroendocrine (NE) subtypes toward non-NE lesions and from non-NE landscape toward NE subtypes were both detected.

**Conclusions:**

Although the molecular landscape of SCLC LN metastases largely resembles that of the tumor of origin, key differences exist in terms of DLL3 and NEUROD1 expression, and in subtype distribution. These diagnostic pitfalls should be considered when establishing the tumors’ molecular profile for future clinical trials solely based on LN biopsies.

## Highlights

• Comparative expression analysis reveals considerably higher DLL3 levels in SCLC primary tumors than in lymph node metastases.
• Lymph node metastases had significantly higher NEUROD1 expression compared with primary lesions.
• Phenotype switching from neuroendocrine subtypes toward non-neuroendocrine lesions and vice versa were both detected.

## Introduction

Small-cell lung cancer (SCLC) is a highly aggressive disease, accounting for 13%-15% of all lung cancer cases.^1^^,^^2^ SCLC tumorigenesis strongly correlates with carcinogen exposure through tobacco use and often exhibits a high mutational burden. Furthermore, biallelic inactivation of the tumor suppressor genes *TP53* and *RB1* are commonly observed.^3^, ^4^, ^5^, ^6^ Pulmonary neuroendocrine (NE) cells and certain epithelial cells are thought to be progenitors of SCLC, while these tumors can also arise from pretreated, epidermal growth factor receptor (EGFR)-mutated or anaplastic lymphoma kinase (ALK)-translocated lung adenocarcinomas through transdifferentiation in selected cases.^7^^,^^8^

Due to the increased proliferation rate and great metastatic potential of SCLC, the majority of patients are diagnosed when the tumor has disseminated outside the chest, and surgical resection is no longer a feasible therapeutic option.^2^ In fact, nearly 85% of patients show extensive-stage (ES) disease with metastases to the regional lymph nodes, the brain, the liver, or the bones.^4^ The dismal 5-year survival rate of 1% for ES patients underlines the aggressive nature of this lethal malignancy for which no relevant therapeutic breakthrough has been achieved in nearly 40 years.^2^^,^^9^ Indeed, the standard of care of SCLC patients has consisted of platinum-based chemotherapy in combination with the topoisomerase inhibitor etoposide (EP) for decades.^10^ In recent years, the addition of immunotherapy targeting the PD-1/PD-L1 axis to the EP backbone has slightly increased overall survival (OS) rates and tripled the 3-year outcomes for a small group of long-term responders.^11^, ^12^, ^13^ Nevertheless, existing intertumoral heterogeneity in SCLC tumors and the lack of targetable driver mutations make it difficult to treat SCLC patients effectively.^2^^,^^14^

Although SCLC is still regarded as a homogenous disease in the clinic, preclinical advancements in the past years have uncovered a heterogeneous underlying molecular profile with predominant molecular subtypes. These biologically distinct subtypes are based on the relative expression of key transcription factors ASCL1 (SCLC-A subtype), NEUROD1 (SCLC-N subtype) and POU2F3 (SCLC-P subtype), and on inflammatory characteristics (SCLC-I subtype).^14^, ^15^, ^16^ Of note, the latter was previously classified according to the expression of the YAP1 transcription regulator, but subsequent validating studies, including ours, could not distinguish a unique SCLC-Y subtype in representative human tissue specimens.^16^, ^17^, ^18^ Instead, these tumors are characterized by the low expression of the aforementioned transcription factors and show an immune cell-rich inflamed phenotype.^14^^,^^16^ Roughly 40%-50% of tumors belong to the NE-high subtype SCLC-A with positive NE marker expression.^4^^,^^15^ Importantly, elevated ASCL1 expression has been associated with poor prognosis in surgically treated SCLC and lung adenocarcinoma patients.^19^, ^20^, ^21^ Meanwhile, SCLC-N tumors demonstrate a lower NE expression profile than SCLC-A. Recent studies suggest that combined SCLC-AN subtypes with elevated ASCL1 and NEUROD1 expression also exist. This subtype is considered to represent an intermediate state during subtype switching, with ASCL1 expression being an obligatory precursor for SCLC-N development.^4^^,^^17^^,^^18^ SCLC-P tumors have been classified as lesions with non-NE phenotype. Notably, these tumors originate from the pulmonary tuft cells, and they have been linked with improved OS in surgically resected patients.^22^

We previously showed that the NE pattern of lymph node (LN) metastases might not always reflect that of the primary tumor in SCLC.^23^ Similarly, Stewart et al. observed dynamic heterogeneity when analyzing the expression of potentially targetable markers in SCLC such as the cell surface protein delta-like ligand 3 (DLL3).^24^ In this study, we aimed to assess the molecular discordance between primary tumors and corresponding LN metastases in light of subtype distribution, and expression of clinically relevant proteins.

## Materials and methods

### Study population

In this study, we included 32 LN metastatic SCLC patients who underwent surgical resection between 1978 and 2013 at the National Koranyi Institute of Pulmonology, Budapest, Hungary. Primary tumors and their corresponding LN metastases were collected at the time of surgery. Clinicopathological data including sex, age at the time of surgery, smoking history, surgical parameters, and survival data were retrospectively retrieved from medical records and/or the Central Statistical Office of Hungary. The study was conducted in accordance with the guidelines of the Helsinki Declaration of the World Medical Association and with the approval of the national-level ethics committee (Hungarian Scientific and Research Ethics Committee of the Medical Research Council, ETT-TUKEB-7214-1/2016/EKU). Patient identifiers were removed after clinical data collection to ensure patient anonymity. Thus, patients cannot be directly or indirectly identified. Due to the retrospective nature of the study, the need for individual informed consent was waived.

### Treatment

Therapeutic approaches were conducted in accordance with the contemporary National Comprehensive Cancer Network (NCCN) guidelines.^25^ Surgery consisted of anatomic resection (including lobectomy or pneumonectomy) or wedge resection. If recommended, adjuvant chemotherapy comprising a platinum-etoposide doublet regimen or a combination of cyclophosphamide, epirubicin, and vincristine (CEV) was also administered.^14^ None of the included patients in the current study received immunotherapy.

### RNA expression analysis

Formalin-fixed paraffin-embedded (FFPE) tissue samples of primary tumors and corresponding LN metastases were macrodissected and subjected to molecular analysis. Since extraction-free RNA measurements were carried out, no RNA extraction of the samples was necessary. Slides were scraped and lysed at 12.5 mm^2^/35 μl for all samples. RNA expression data were obtained using the HTG EdgeSeq Targeted Oncology Biomarker Panel (HTG Molecular Diagnostics Inc., Tucson, AZ), capable of simultaneous, quantitative detection of 2560 genes associated with tumor biology. This is a targeted RNA expression assay that is generated via nuclease protection and consists of the hybridization of target RNA to a DNA probe, followed by treatment with single-strand nuclease. Notably, the used panel was explicitly designed to identify known cancer-specific therapeutic targets and drug response markers. Appropriate negative and positive controls were used for assay validation.

### Tissue processing and immunohistochemistry (IHC)

Before enrolment, all hematoxylin–eosin (H&E)-stained slides were re-evaluated by a board-certified pulmonary pathologist to confirm the diagnosis of SCLC. Quality control of older blocks was achieved within the framework of our previous study^18^ by performing confirmatory IHC staining with routinely used diagnostic antibodies. TMA construction was carried out at the University of Colorado, Denver (Aurora, CO), as previously described.^26^ Notably, two 1-mm punches of tissue were taken from each donor tissue block and placed into a recipient paraffin block in a positionally encoded array format (MP10 1.0-mm tissue punch on a manual TMA instrument, Beecher Instruments, Sun Prairie, WI). Tissue cores were retrieved from the most viable tumor areas as defined on H&E-stained slides. In addition to subtype markers (ASCL1, NEUROD1, POU2F3, and YAP1), the expression of the following clinically relevant proteins was evaluated: CD47, c-myc, l-myc, DLL3, Ezh2, LSD1, mTOR, PD-L1, and PIK3. Concerning IHC staining, after deparaffinization and rehydration of the 4-μm thick sections, slides were heated for 20 min in either 10 mM citrate buffer (pH 6.0) or in 10 mM Tris-EDTA buffer (pH 9.0) according to antibody protocols. To reduce background staining, slides were incubated in a 0.3% H_2_O_2_ solution. Signal amplification was carried out according to the manufacturer’s recommendation of the Novolink^TM^ Polymer Detection System kit from Leica Biosystems (RE7150-K; Wetzlar, Germany), followed by antibody incubation at room temperature for 1 h. The used antibodies are listed in Supplementary Table S1, available at https://doi.org/10.1016/j.esmoop.2025.104514. Antibody binding was detected with the ImmPACT DAB substrate kit from Vector Laboratories (NC9567138; Newark, CA). Nuclei were counterstained using hematoxylin. All antibodies used for IHC analyses were validated using appropriate tissue controls selected based on specific protein expression data of the Human Protein Atlas (HPA),^27^ and on the manufacturers’ recommendations. Appropriate tissue controls corresponding to each antibody are listed in Supplementary Table S1, available at https://doi.org/10.1016/j.esmoop.2025.104514. IHC-labeled and H&E-stained slides were digitally scanned using PANNORAMIC 250 Flash III (3DHISTECH Ltd., Budapest, Hungary); sections were evaluated with CaseViewer 2.4 (3DHISTECH Ltd., Budapest, Hungary). Since a reduction of immunosignal intensity may occur in some FFPE samples with time,^28^^,^^29^ we focused solely on determining the percentage of positive cells during the pathological evaluation rather than assessing the H-score—which includes the semi-quantitative measurement of both staining intensity and the percentage of positive cells. Specifically, we determined the percentage of positive tumor cells in at least 20 randomly selected areas at ×20 and ×40 magnification. To minimize subjectivity, two experienced pulmonary pathologists evaluated the IHC stainings independently of each other, and their average scores were used for further statistical calculations. Importantly, if their results differed by >20%, a third pulmonary pathologist was also involved in the final scoring.

### Statistical analysis

RNAseq data was quantified as ‘normalized CPM (counts per million)’, measuring the number of sequenced fragments aligning to a specific gene out of 1 million sequenced reads, normalized with the length of the gene. The initial analysis of RNAseq results was carried out automatically by the default processing pipeline utilized by the HTG EdgeSeq Targeted Oncology Biomarker Panel. When assessing batch effects, neither principal component analysis (PCA) nor t-distributed stochastic neighbor embedding (t-SNE) shows a significant clustering tendency among the samples (Supplementary Figure S1, available at https://doi.org/10.1016/j.esmoop.2025.104514), indicating that the batch effects are not substantial. Furthermore, since samples were analyzed in pairs (primary tumor versus LN metastasis), both of which were prepared in the same year using the same methodology, any time-related and technical batch effects cancel out throughout these comparisons. Therefore, subsequent investigations were based on the normalized CPM values. Further batch effect assessment steps are detailed in Supplementary Materials and Methods, and in Supplementary Figures S1 and S2, available at https://doi.org/10.1016/j.esmoop.2025.104514.

Fold changes (FC) between the expression levels of LN metastases and primary tumors were calculated by dividing the expression (normalized CPM or IHC positivity) of the given gene/protein in the LN sample by the expression level of the same gene/protein in the primary tumor sample of the same patient. The log_2_-transforms of these ratios are shown in the figures.

Expression levels across different sample origins (primary tumors and LN metastases) were compared with *t*-tests and Wilcoxon signed rank tests. Correction for multiple testing was carried out with the Holm method. Correlations between expression levels measured with the two experimental protocols (IHC staining versus RNAseq) were assessed by calculating the Pearson correlation coefficients and the corresponding *P* values. Hierarchical clustering of samples based on expression levels was carried out with the ComplexHeatmap R package (version 2.10.0). The distance matrix was calculated using Eucledian distance measure and the dendrograms were created using the ward.D clustering method. To determine whether primary tumors and LN metastases are linearly separable in the expression space of the genes and proteins of interest, PCA was carried out with the factoextra R package (version 1.0.7). Before PCA, expression levels were centered and scaled to have zero mean and unit variance. All statistical analyses were carried out in R version 4.2.1 (R Foundation for Statistical Computing, Vienna, Austria).

## Results

### Patient and sample characteristics

A total of 32 surgically treated patients with histologically confirmed SCLC were included in this retrospective study. The median age of included patients was 58 years (range 34-78 years). Twenty-two patients were male and all included patients had a Caucasian background. Median OS was 20.7 months, whereas the median disease-free survival of the study population was 14.9 months.

### Comparative expression analysis of molecules of interest

**Figure 1:**
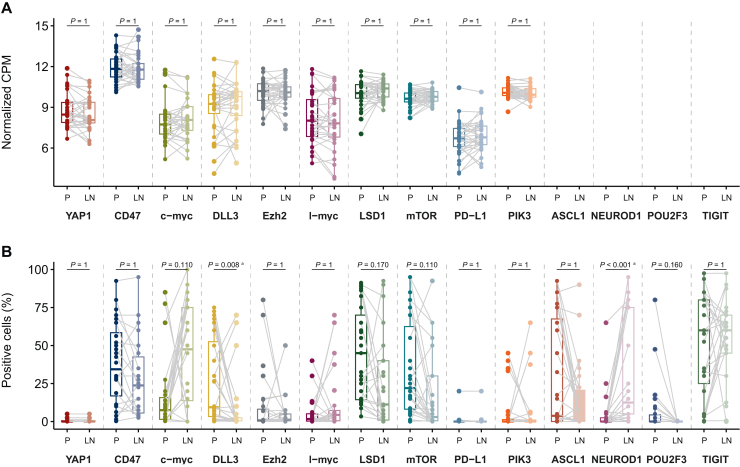
**Expression pattern of genes (A) and proteins (B) of interest in SCLC primary (P) tumors and lymph node (LN) metastases.** The *P* values were calculated by using *t*-tests (A) and paired Wilcoxon signed rank tests (B). For multiple correction testing, the Holm method was applied. DLL3 expression was significantly higher in the primary tumors than in the LN metastases, whereas NEUROD1 expression was significantly lower in the primary lesions (versus LN metastases). CPM, counts per million. ^a^Results significant after correction.

**Figure 2:**
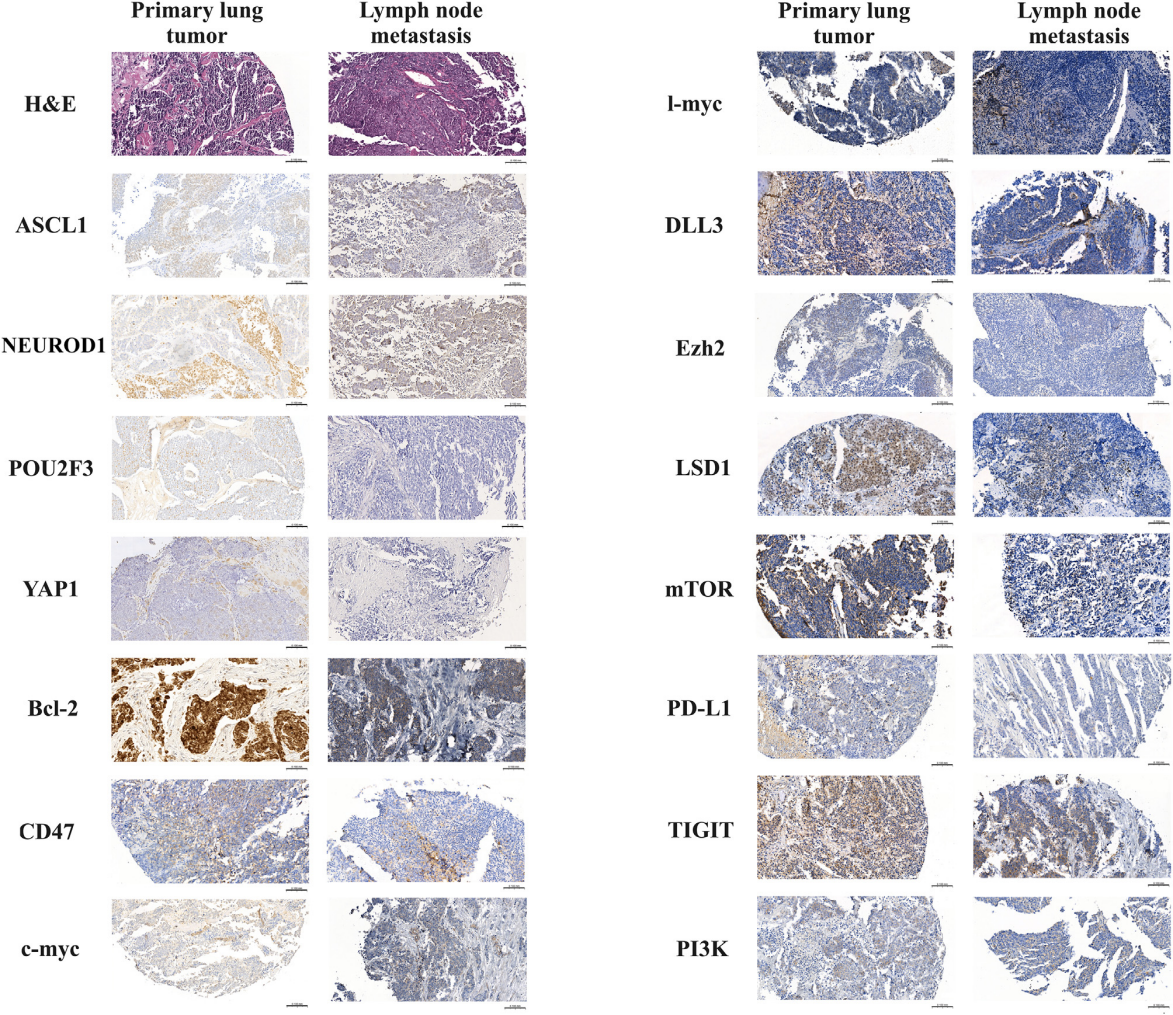
**Immunohistochemistry stainings of formalin-fixed, paraffin-embedded primary and lymph node (LN) metastatic small-cell lung cancer samples with subtype markers and other proteins of interest.** Given that the expression patterns of the markers of interest in LN metastases may differ from those in the primary tumors, the representative tissue sections from LN metastases and primary tumors may not necessarily originate from the same patient. The representative images were captured with a ×20 objective lens. The positive cells were visualized with 3-3′ -diaminobenzidine (DAB), and the nuclei were labeled with hematoxylin. The top left row corresponds to the routine hematoxylin–eosin (H&E) staining.

An overview of expression differences concerning the genetic landscape of primary tumors and corresponding LN metastases based on RNAseq data is shown in Supplementary Figure S3, available at https://doi.org/10.1016/j.esmoop.2025.104514. After applying multiple testing corrections with log_2_-FC <0, the top five genes statistically significantly downregulated in LN metastases (versus primary tumors) were *PGC*, *FIGF*, *ROS1*, *CFTR*, and *TMPRSS2*. The molecules of interest for the current study showed no significant differences in their RNA expression profile when assessing the primary tumors and their corresponding LN metastases (Figure 1A and Supplementary Figure S3, available at https://doi.org/10.1016/j.esmoop.2025.104514). Next, we examined whether the predefined molecules differ in their expression pattern at the protein level, as defined by IHC. As shown in Figure 1B, DLL3 expression was significantly higher in the primary tumors than in the LN metastases (*P* = 0.008). Likewise, mean CD47, LSD1, mTOR, and POU2F3 expression was also higher in the primary tumors compared with their LN counterparts, yet these differences did not reach statistical significance. In contrast, NEUROD1 expression was significantly lower in the primary tumors (versus LN metastases, *P* < 0.001), whereas c-myc expression was nonsignificantly lower in these lesions. Representative IHC images of each investigated marker and their expression levels concerning the primary tumors and LN metastases are shown in Figure 2 and in Supplementary Figure S4, available at https://doi.org/10.1016/j.esmoop.2025.104514.

### Correlation between expression levels as defined by RNA expression analysis and IHC

**Figure 3:**
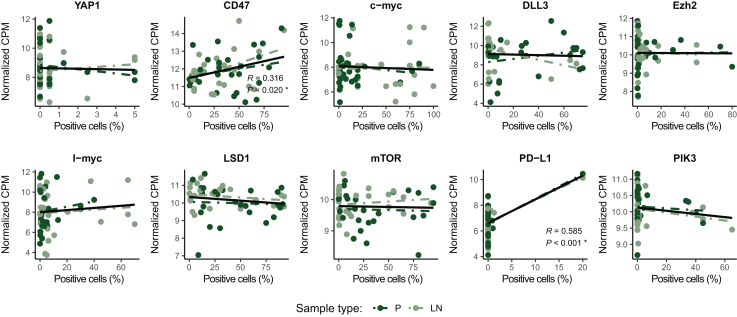
**Correlation of protein and gene expression data as defined by immunohistochemistry and RNA expression analysis.** Colors indicate sample type (P: primary tumor, LN: lymph node metastasis). *R* indicates Pearson correlation coefficient and *P* shows the corresponding *P* value. CPM, counts per million. ∗Statistically significant results.

In general, a moderate to weak correlation was found between RNA expression data and IHC outcomes. YAP1, c-myc, DLL3, EZH2, l-myc, LSD1, mTOR, and PIK3 expression did not correlate between the two datasets. Nevertheless, a moderate positive correlation was detected in the case of both CD47 and PD-L1 expression (R = 0.316, *P* = 0.020 and R = 0.585, *P* < 0.001, respectively; Figure 3).

### Hierarchical clustering and principal component analysis of expression levels

**Figure 4:**
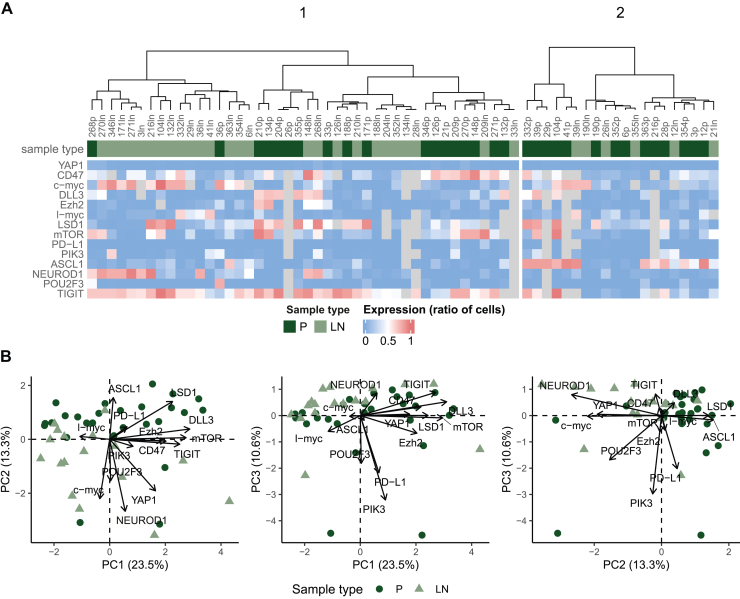
**Hierarchical clustering (A) and principal component (PC) analysis (B) of primary (P) tumors and lymph node (LN) metastases based on the tumor cell expression of proteins of interest.** (A) The color of the bars on the top of the heatmap indicates the sample type. Heatmap colors correspond to immunohistochemistry (IHC) staining positivity defined as the ratio of positive cells. The hierarchical clustering of samples cannot effectively differentiate between primary tumors and LN metastases based on the expression pattern of proteins of interest. (B) The panels show the samples projected to the subspaces spanned by the first three PCs. Colors indicate sample type. The percentage of variance explained by each PC is indicated in the axis labels. Arrows indicate the direction of the protein expression levels in the subspaces. IHC expression levels of the investigated proteins alone are not sufficient to truly distinguish between samples of different origins.

Next, we investigated whether the protein expression pattern can discriminate between primary tumors and LN metastases. As shown in Figure 4A, although cluster analysis differentiated two distinct subgroups with divergent protein expression profiles, these clusters did not conclude with the site of origin. Interestingly, the transcription factor YAP1 consistently showed low expression levels irrespective of the sample type, whereas TIGIT, an immune checkpoint that plays a pivotal role in immune suppression, was overexpressed in the vast majority of both primary and LN metastatic tumors. Of note, mTOR and DLL3, both representing emerging target/binding proteins in SCLC, were differentially expressed across the tumors. With a few exceptions, PD-L1 expression was generally low. Principal component (PC) analysis (Figure 4B) of the IHC results showed that 47.4% of the variance in the data is explained by the first three PCs. Of note, when projecting the data points (samples) to the space spanned by the first two PCs, there was only a modest separation between primary tumors and LN metastases, suggesting that IHC expression levels of the investigated proteins alone are not sufficient to truly distinguish between samples of different origin. RNA expression data was also unable to classify samples according to their site of origin (Supplementary Figure S5, available at https://doi.org/10.1016/j.esmoop.2025.104514).

### Comparative IHC analysis reveals molecular subtype change between primary tumors and corresponding LN metastases

**Figure 5:**
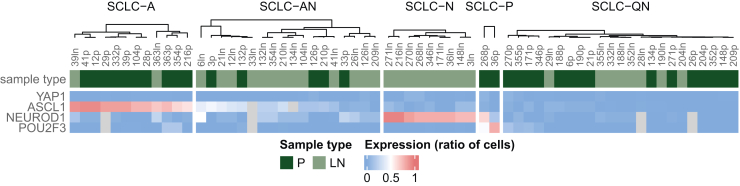
**Molecular subtypes of surgically resected primary tumors and lymph node (LN) metastases as defined by immunohistochemistry (IHC)**. Hierarchical clustering of primary (P) tumors and LN metastases revealed five distinct small-cell lung cancer (SCLC) subgroups: SCLC-A (ASCL1-defined), SCLC-N (NEUROD1-defined), SCLC-AN (combined ASCL1/NEUROD1), SCLC-P (POU2F3-defined), and SCLC-QN (quadruple negative). The color of the bars on the top of the heatmap indicates the sample type. Heatmap colors correspond to IHC staining positivity defined as the ratio of tumor cells showing positive staining. Primary tumors and their corresponding LN metastases frequently cluster in different subgroups.

Differential expression analysis of subtype-defining proteins distinguished five major SCLC subgroups across the included tumor specimens. As shown in Figure 5, besides SCLC-A (ASCL1-defined), SCLC-AN (combined ASCL1/NEUROD1), SCLC-N (NEUROD1-defined), and SCLC-P (POU2F3-defined), the previously proposed quadruple negative SCLC subtype (SCLC-QN) was also identified in our cohort. This latter subgroup was characterized by low expression of all four (ASCL1, NEUROD1, POU2F3, and YAP1) transcription regulators. No unique YAP1-defined subtype (SCLC-Y) could be distinguished by cluster analysis. The majority (61%) of samples had an NE phenotype (SCLC-A, SCLC-AN, and SCLC-N). Next, we investigated whether clustering of the primary tumor samples is indicative of the subtypes of subsequent LN metastases or if they markedly differ in their expression profiles concerning subtype-defining proteins. In this context, we found that the molecular subtype of the corresponding LN metastases might not mimic that of the primary tumor as primary lesions and their LN metastases frequently clustered in different subgroups (Figure 5 and Supplementary Figure S6, available at https://doi.org/10.1016/j.esmoop.2025.104514). Altogether, a change in molecular subtyping was detected in 21 cases. Most commonly, SCLC-QN primary tumors exhibited SCLC-AN (*n* = 5) or SCLC-N (*n* = 5) subtypes in their corresponding LN metastases. Other changes included SCLC-A to SCLC-AN (*n* = 4), SCLC-N (*n* = 1), or SCLC-QN (*n* = 3). Interestingly, the majority of SCLC-AN primary tumors retained their molecular phenotypes during metastatic spread, and subtype switching occurred in only one patient (to SCLC-N). None of the primary tumors had an SCLC-N subtype, while it was the second most frequent subtype in LN. Both primary tumors with SCLC-P phenotype gave rise to SCLC-N LN metastases.

## Discussion

SCLC is an aggressive disease with a high propensity for metastasis, particularly to regional LNs.^30^ Interestingly, the underlying landscape of the primary tumors does not always resemble the molecular features seen in LN metastases, suggesting that the expression profile of therapeutic targets and subtype markers might differ across sites of origin.^31^ Since such heterogeneity can influence consequent treatment choice, this study investigated the differential expression patterns of molecules of interest between primary tumors and their matched LN metastases using IHC and RNA sequencing.

Examining the gene expression profile of primary tumors and their corresponding LN metastases through RNAseq revealed no statistically significant differential expression of the investigated molecules. In contrast, IHC staining enabled a moderate distinction between the two sites. Specifically, the proteins DLL3 and NEUROD1 showed varying expression in primary tumors compared with their matched LN metastases. Thus, staining for the proteins through IHC detected differences in protein expression that could not be validated via RNAseq. Nevertheless, IHC staining measures the relative protein expression in tumor cells whereas the analysis of RNA gene expression does not discriminate entirely between gene expression levels in tumor cells and surrounding stromal cells. Indeed, studies in non-small-cell lung cancer have reported a ∼80% concordance between IHC and RNAseq results.^32^ As for the differential analysis of the general genomic landscape of primary tumors and LN metastases, five genes (*PGC*, *FIGF*, *ROS1*, *CFTR*, *TMPRSS2*) showed significant downregulation in LN metastases after multiple testing correction. These proteins are either cancer-related or exhibit lung-specific expression profiles. Among others, *ROS1* is a commonly known proto-oncogene that is highly expressed in tumor cells and its mutations have been previously described in lung cancer.^33^, ^34^, ^35^

In our study, IHC analysis revealed that DLL3 was significantly overexpressed in primary tumors compared with matched LN metastases. This downregulation of DLL3 in LN metastases can be attributed to differences in the regulation of gene expression on a post-transcriptional, translational, or post-translational level.^36^ The cell surface protein DLL3 is an oncogene expressed in >80% of SCLC tumors and promotes both cell proliferation and migration.^37^ DLL3 serves as a potent inhibitor of Notch signaling and directs the degradation of protein members in the same pathway. Overexpression of this oncogene has been associated with a worse prognosis for patients.^38^ Importantly, as DLL3 is a surface protein found exclusively on SCLC tumors and is absent in healthy lung tissue, DLL3 has become a relevant therapeutic target and is currently being investigated in multiple clinical trials in SCLC.^39^ Indeed, allogenic chimeric antigen receptor (CAR)-T cells targeting DLL3 showed high efficacy in systemic *in vivo* mouse models.^40^ Furthermore, the bispecific T-cell engager (BiTE) tarlatamab (AMG 757) has been recently granted accelerated Food and Drug Administration (FDA) approval for pretreated, recurrent SCLC patients.^41^ Results of the open-label, multicenter, phase II trial (DeLLphi-301) reflected both drug efficacy (overall response rate 40% for 10 mg versus 32% for 100 mg) and manageable tolerability, with median OS rates of 14.3 months.^42^ Since recurrent SCLC patients are often heavily pretreated and prone to the development of resistance,^43^ results achieving both efficacy and safety are encouraging and stand in contrast to those of clinical trial outcomes for the antibody drug conjugate rovalpituzumab tesirine (Rova-T) which was discontinued in a clinical trial due to the toxicity profile.^44^^,^^45^ Although drugs targeting DLL3 are currently administered to metastatic SCLC patients irrespective of their DLL3 expression status, investigation of patient-specific DLL3 expression might change current clinical practices in the years to come. In this context, our results suggest that assessment of the primary tumor’s DLL3 is crucial and of diagnostic importance, as the corresponding LN metastases might not entirely mimic the DLL3 expression of the lung lesion. The transcription factor NEUROD1 was the second protein showing significant differential protein expression after multiple testing correction between primary tumors and LN metastases according to IHC. Both the field at large and our group have demonstrated this protein to be a potential subtype-defining marker with prognostic implications on survival.^9^^,^^15^^,^^18^ Hence, further investigations on differential expression and pathways of proteins such as NEUROD1 will be useful to the advancement of subtyping efforts for SCLC.

Previous studies have demonstrated considerable inter- and intratumoral heterogeneity in SCLC subtype expression across samples.^17^, ^18^, ^19^ In our study, hierarchical clustering distinguished five distinct subtypes (SCLC-A, -AN, -N, -P, and -QN) in both primary and LN metastatic lesions. No distinct YAP1-defined subtype could be distinguished in our cohort, which is consistent with the findings of multiple independent analyses (including those from the investigators originally proposing YAP1 as a candidate subtype)^14^^,^^17^^,^^46^, ^47^, ^48^ This lack of a unique SCLC-Y subtype might be attributed to tumor heterogeneity,^17^ therapy-induced plasticity,^49^^,^^50^ and technical controversies regarding the original classification scheme.^51^ Indeed, due to temporal evolution influenced by Notch activity and to the selection of subclones that exhibit intrinsic or acquired resistance,^38^^,^^50^ YAP1 expression is more prevalent in end-stage/relapsed tumors than in earlier stage/untreated SCLCs examined within the framework of the current study. Additionally, it is important to note that the subtype-defining potential of YAP1 has recently been fundamentally questioned as multiomic reassessment of the presumed YAP1-high SCLC cell lines (that served as the basis for the original classification scheme) revealed that nearly all of these tumors are actually previously unrecognized SMARCA4-deficient undifferentiated tumors or other types of malignancies rather than SCLC.^51^ Interestingly, when examining the matched specimens according to their dominant molecular subtype, we noted considerable changes in subtype distribution in paired LN metastases (versus their primary tumor counterparts). Notably, a subtype change from SCLC-A to subtypes with less neuroendocrine features (SCLC-AN or SCLC-N, respectively) was frequently observed. This might be due to the tumor’s natural temporal evolution, as SCLC-A has been characterized as a necessary precursor for the neuronal subtype SCLC-N.^52^^,^^53^ In this process, endogenous activation of Notch signaling results in the loss of NE differentiation, thereby facilitating the transition from an NE-high to NE-low phenotype and potentially on to a non-NE landscape.^54^^,^^55^ This hypothesis is further supported by the fact that, in line with others, we also distinguished an SCLC-AN subtype. This combined subtype might represent a transition state from an NE-high to a more NE-low phenotype. Of note, although none of the included patients received neoadjuvant systemic therapy before surgery, predominant subtype expression is also likely to be influenced by a response to systemic treatment and various processes during metastatic spread.^16^^,^^18^^,^^23^ Interestingly, our study also showed changes from less or no NE (SCLC-P and SCLC-QN) phenotypes to NE features (SCLC-AN, SCLC-N, or SCLC-A) when assessing the primary tumors and their LN metastases. Although these primary lesions exhibited predominantly non-NE landscapes, given the heterogeneous nature of the tumor,^18^ SCLC cells with NE characteristics might also have been present. Since NE tumor cells are more aggressive than those with low or non-existing NE features, they might be the primary source of LN metastasis, forming NE-high lesions.^14^

Some limitations in our study must be addressed. Firstly, although the study material is unique considering the scarcity of surgically resected tumor specimens in SCLC, the sample size remains moderate for expression analysis studies. Therefore, additional validation studies are warranted. Secondly, while tissue samples for RNA expression analysis were retrieved from the most viable tumor areas through careful macrodissection, they might still contain underlying nonmalignant tumor stroma compartments, slightly impacting the RNAseq outcomes. Of note, this latter limitation only applies to RNAseq data, as IHC staining was carried out solely on tumorous cells. Lastly, IHC expression levels on TMA samples might be biased by intratumoral heterogeneity; however, to partly overcome this issue, all TMAs included in our study contained two separate tissue cores originating from the same tumor.

### Conclusion

The current study attempts to provide early insights into molecular changes during metastatic spread by analyzing a unique cohort of surgically resected primary SCLCs and their corresponding LN metastases in a comparative manner. Although the molecular profile of the LN metastases resembles that of the tumor of origin, key differences exist in terms of DLL3 and NEUROD1 expression. Accordingly, DLL3 expression was significantly higher in primary tumors than in corresponding LN metastases, whereas NEUROD1 was expressed to a greater extent in LN lesions. Furthermore, we also show that the molecular subtype of the LN metastases does not ubiquitously mirror the molecular classification of the primary tumor, suggesting potential subtype changes during metastatic spread through the lymphatic system. Phenotype switching from NE subtypes toward non-NE lesions and from non-NE landscape toward NE subtypes were both detected. These results raise awareness of potential diagnostic pitfalls when establishing the in-depth molecular profile and molecular subtype of SCLCs using LN biopsies.
