# Supplementary figures and images for: Comparative profiling of surgically resected primary tumors and their lymph node metastases in small-cell lung cancer
Source: ESMO Open. 2025 Mar 18;10(4):104514. doi: 10.1016/j.esmoop.2025.104514 (PMC11964634; doi:10.1016/j.esmoop.2025.104514)

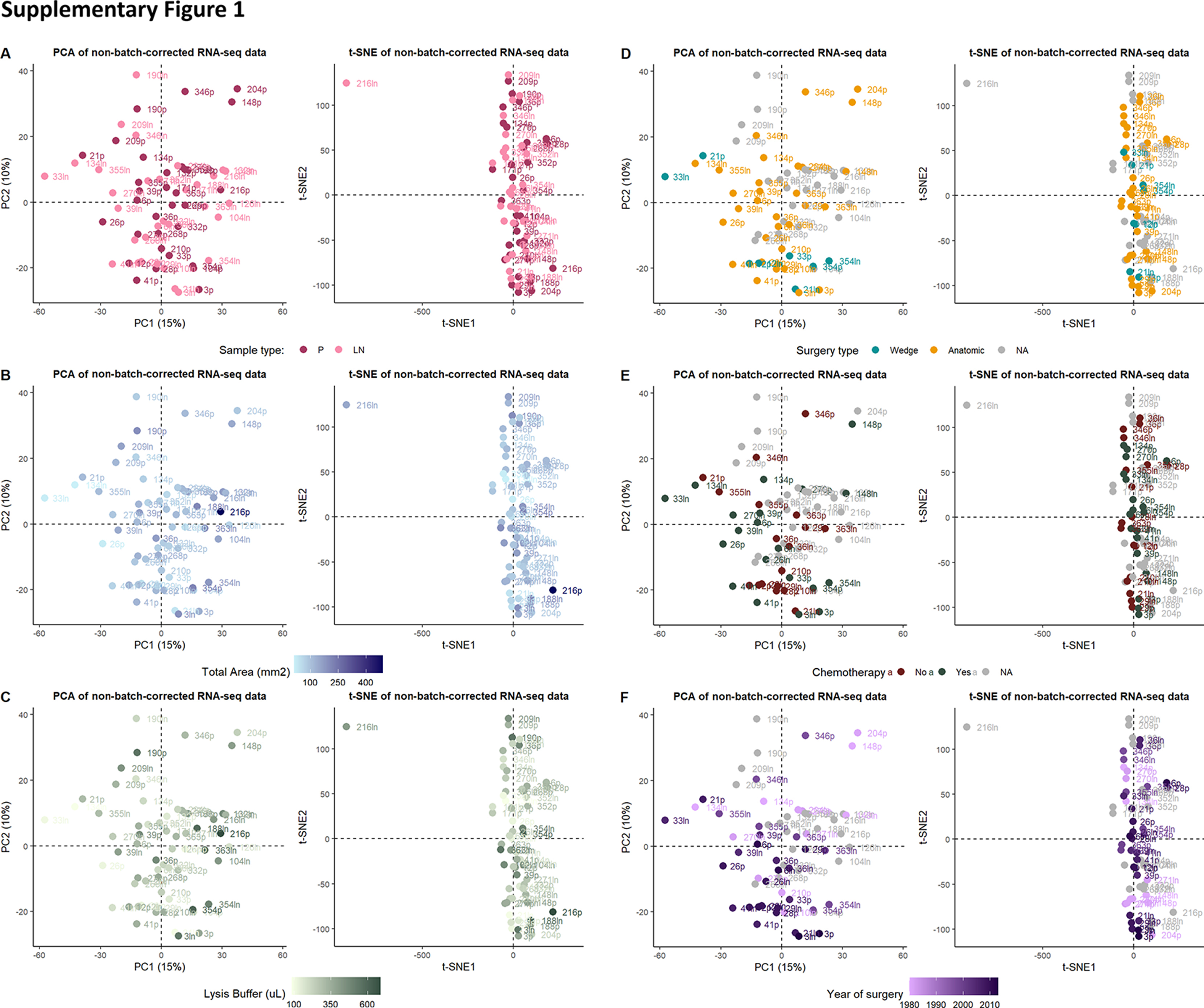

Supplement: Supplementary Figure 1 [file figs1.jpg]

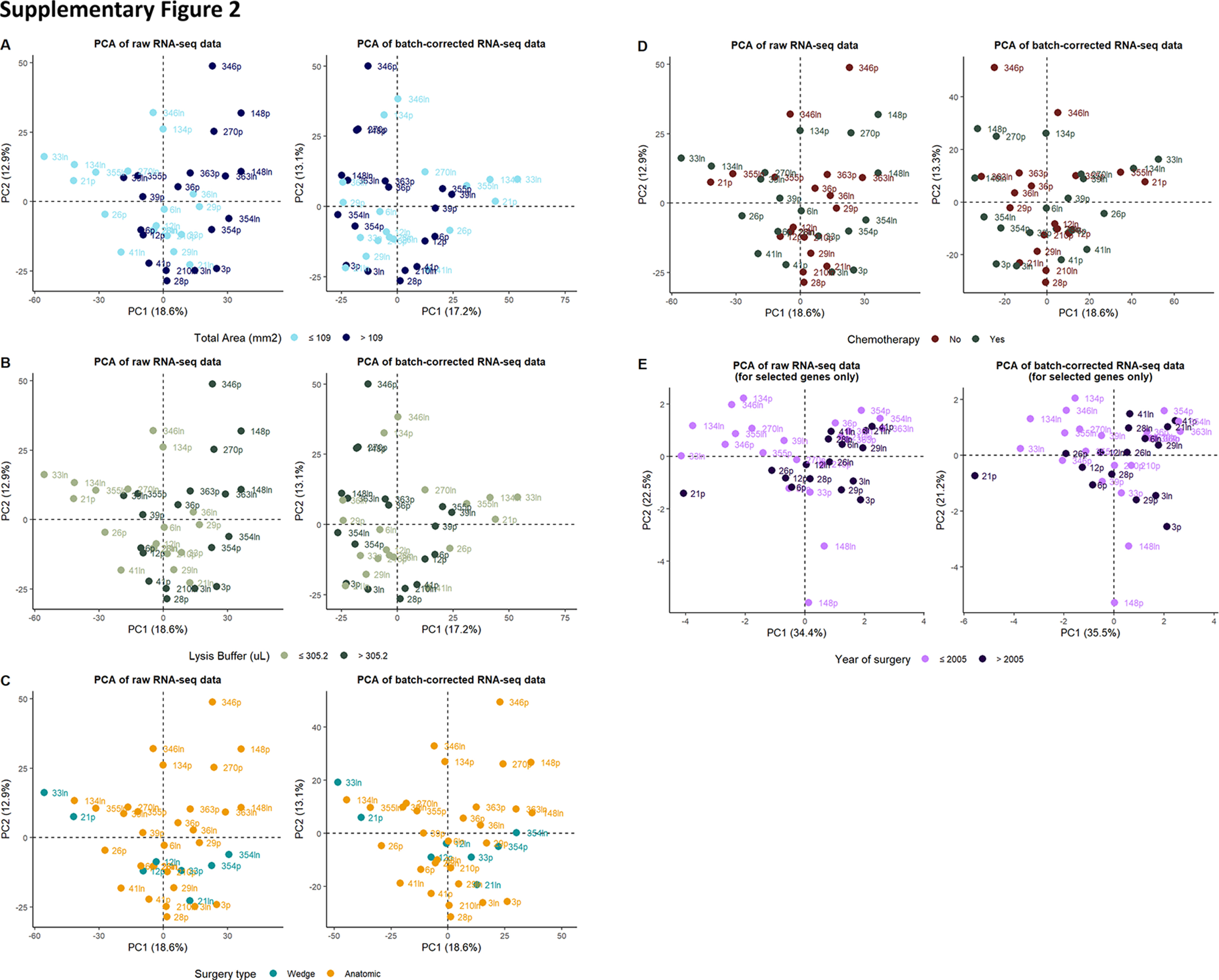

Supplement: Supplementary Figure 2 [file figs2.jpg]

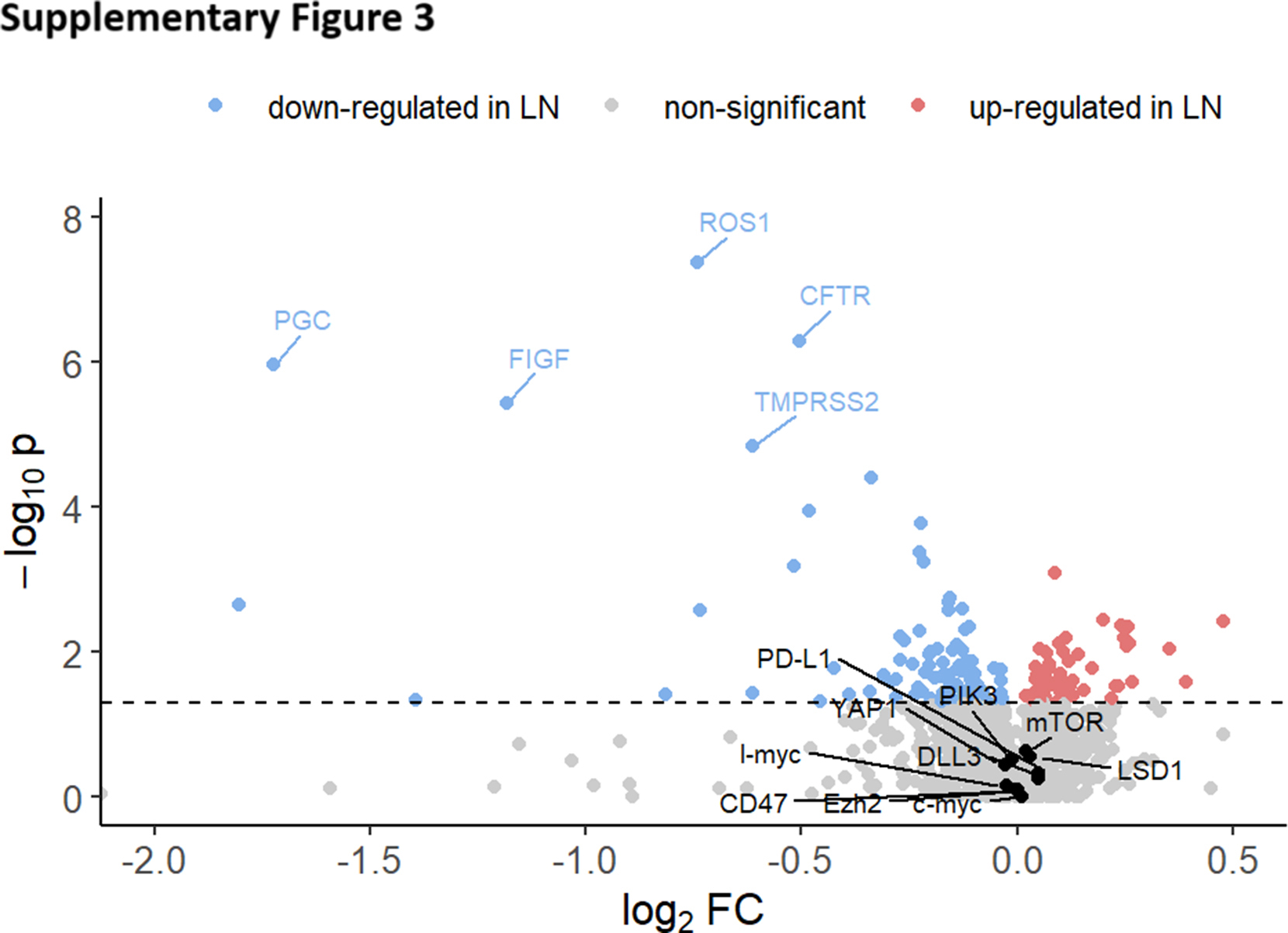

Supplement: Supplementary Figure 3 [file figs3.jpg]

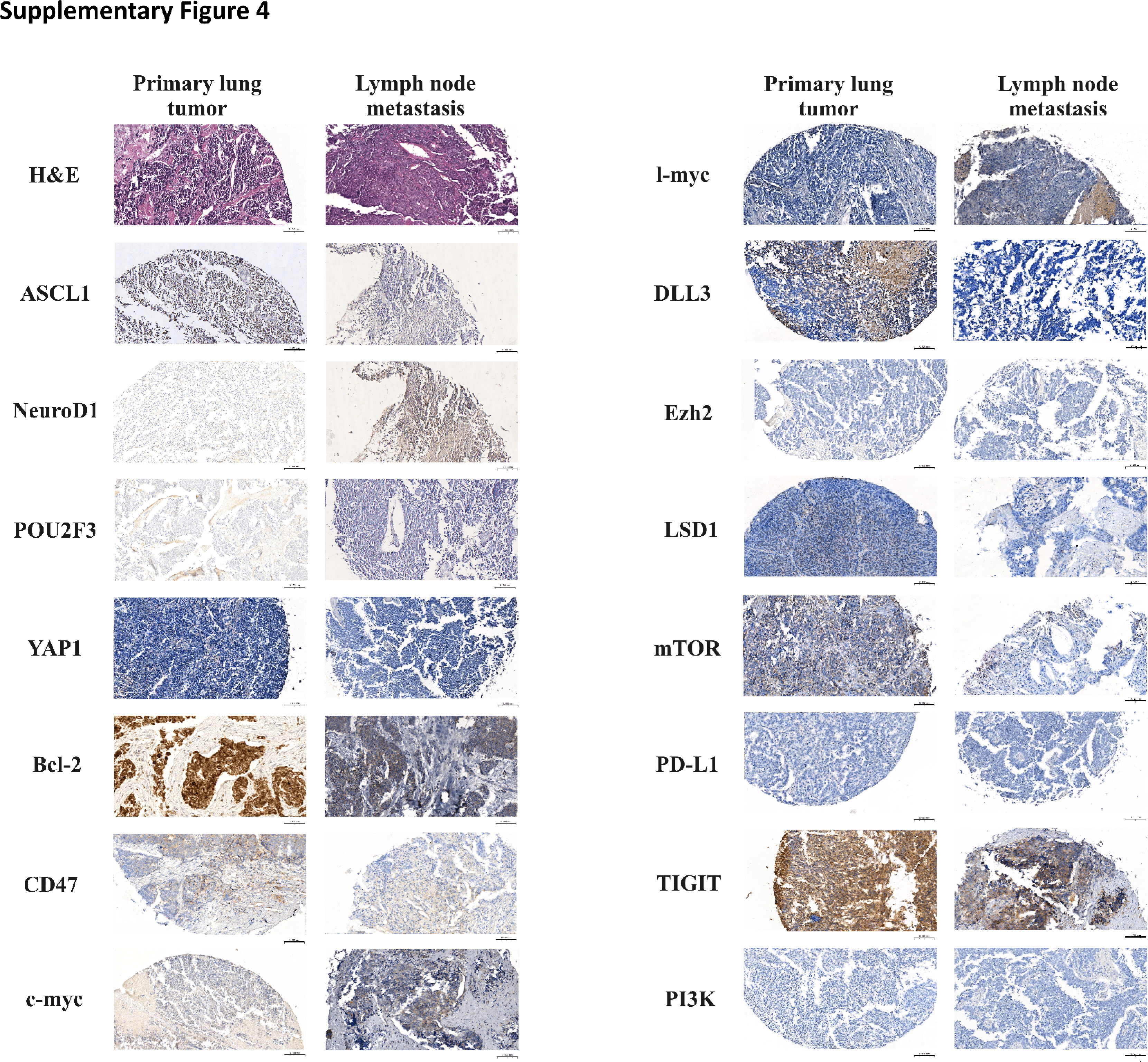

Supplement: Supplementary Figure 4 [file figs4.jpg]

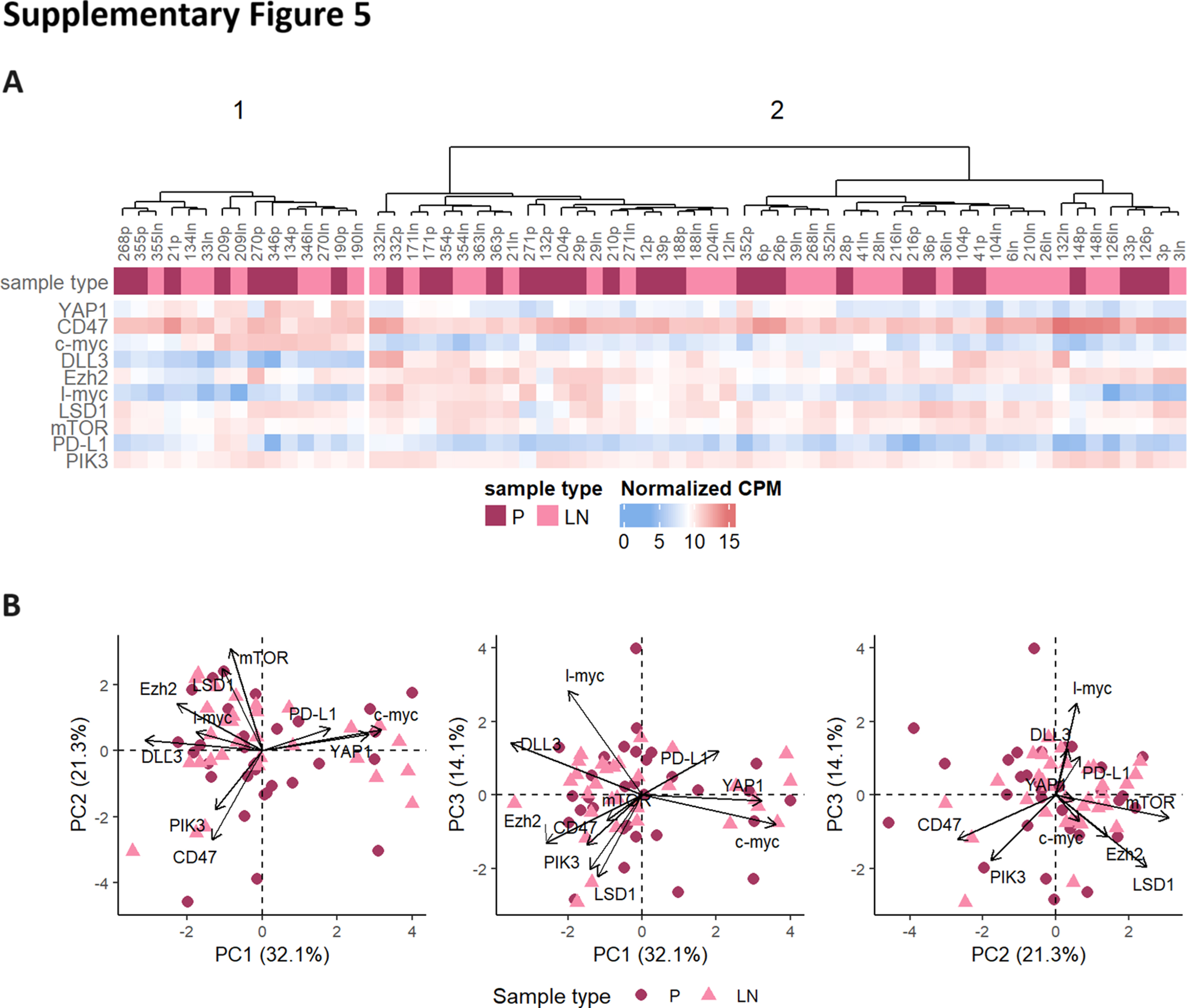

Supplement: Supplementary Figure 5 [file figs5.jpg]

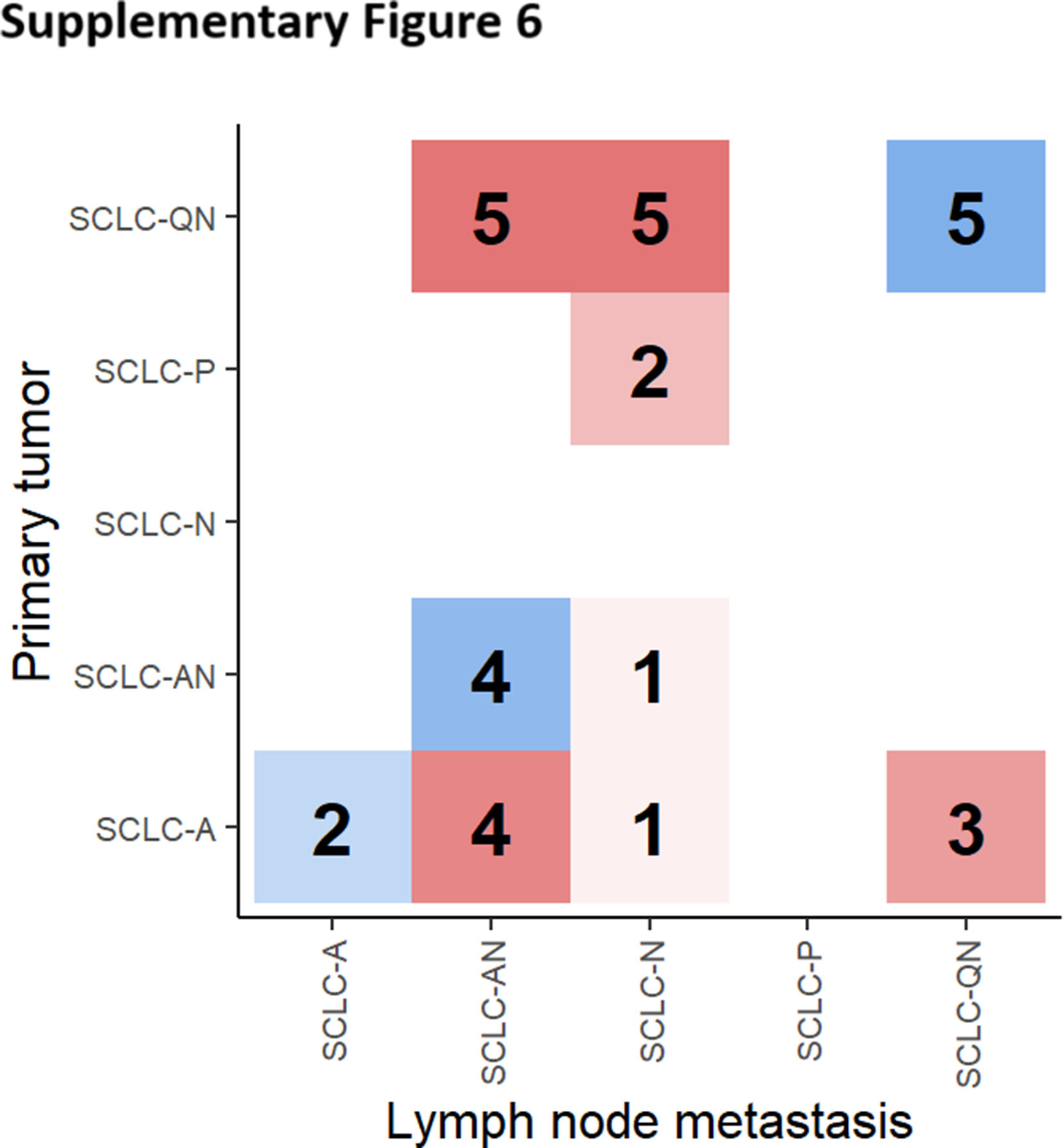

Supplement: Supplementary Figure 6 [file figs6.jpg]
