# Supplementary material for: Comparative profiling of surgically resected primary tumors and their lymph node metastases in small-cell lung cancer
Source: ESMO Open. 2025 Mar 18;10(4):104514. doi: 10.1016/j.esmoop.2025.104514 (PMC11964634; doi:10.1016/j.esmoop.2025.104514)
Supplement: Supplementary Tables [file mmc1.docx]

| Antibody | Company | Catalog nr. | Clone | Host | Dilution | Antigen retrieval | Positive control |
| --- | --- | --- | --- | --- | --- | --- | --- |
| ASCL1 | BD Pharmigen; Berkshire, UK | 556604 | 24B72D11.1 | Mouse | 1:50 | Citrate (pH=6.0) | Brain |
| NeuroD1 | Abcam; Boston, MA, USA | ab213725 | EPR20766 | Rabbit | 1:100 | Tris-EDTA (pH=9.0) | Cerebellum |
| POU2F3 | Santa Cruz Biotechnology; Dallas, TX, USA | cs-293402 | 6D1 | Mouse | 1:100 | Citrate (pH=6.0) | Colon |
| YAP1 | Cell Signaling Technology; Leiden, The Netherlands | 49125 | N/A | Rabbit | 1:200 | Citrate (pH=6.0) | Kidney |
| Ezh2 | Cell Signaling Technology;  Leiden, The Netherlands | 5246S | D29C | Rabbit | 1:50 | Citrate (pH=6.0) | Tonsil |
| DLL3 | Abcam; Boston, MA, USA | ab103102 | N/A | Rabbit | 1:100 | Tris-EDTA (pH=9.0) | Brain |
| PIK3CA | Bioss; Woburn, MA, USA | bs-2067R | N/A | Rabbit | 1:400 | Citrate (pH=6.0) | Brain |
| mTOR | Abcam; Boston, MA, USA | ab32028 | Y391 | Rabbit | 1:400 | Citrate (pH=6.0) | Kidney |
| LSD1 | Abcam; Boston, MA, USA | ab17721 | N/A | Rabbit | 1:200 | Tris-EDTA (pH=9.0) | Kidney |
| TIGIT | Abcam; Boston, MA, USA | ab243903 | BLR047F | Rabbit | 1:100 | Tris-EDTA (pH=9.0) | Tonsil |
| CD47 | Sigma-Aldrich;  St. Louis, MI, USA | HPA044659 | N/A | Rabbit | 1:100 | Tris-EDTA (pH=9.0) | Brain |
| PD-L1 | Abcam; Boston, MA, USA | ab205921 | 28-8 | Rabbit | 1:500 | Citrate (pH=6.0) | Tonsil |
| c-myc | Abcam; Boston, MA, USA | ab32072 | Y69 | Rabbit | 1:100 | Tris-EDTA (pH=9.0) | Brain |
| l-myc | Thermo Fisher Scientific;  Waltham, MA, USA | PA5-41114 | N/A | Rabbit | 1:200 | Tris-EDTA (pH=9.0) | Kidney |
| Bcl-2 | Leica Biosystems;  Wetzlar, Germany | PA0117 | bcl-2/100/D5 | Mouse | 1:100 | Tris-EDTA (pH=9.0) | Tonsil |

**Supplementary table S1.** Antibodies used for immunohistochemistry.
