# Supplementary material for: Comparative profiling of surgically resected primary tumors and their lymph node metastases in small-cell lung cancer
Source: ESMO Open. 2025 Mar 18;10(4):104514. doi: 10.1016/j.esmoop.2025.104514 (PMC11964634; doi:10.1016/j.esmoop.2025.104514)
Supplement: Supplementary Methods [file mmc2.docx]

**Supplementary** **materials and methods**

*Batch effect assessment*

Supplementary Figure 1 shows the PCA and t-SNE results of the non-batch-corrected RNAseq data based on technical parameters, including sample type, total area of the tissue core, used lysis buffer, type of surgery, adjuvant chemotherapy, and year of surgery. Importantly, no evident clustering tendency of the samples can be observed in any of these plots, indicating that the batch effects are not significant. Of note, sample 216ln is an outlier in the t-SNE plot, but this separation is not observed in the PCA plot. To further investigate the issues related to the possible batch effect, we performed batch effect correction using the ComBat function from the sva package. Since neither DESeq2 nor ComBat accepts missing batch information/metadata and data imputation would cause additional artifacts, we applied these analyses only to the samples for which complete metadata was available (n=38). As shown in Supplementary Figure 2, technical variables show no to minimal batch effect even for the raw data, and none of the variables benefit substantially from batch correction. Given that these results indicate minimal batch effects in our dataset, and correction is not feasible in all cases due to missing metadata, we used the raw normalized CPM values for statistical calculations. Furthermore, since samples were analyzed in pairs (primary tumor vs. lymph node metastasis), both of which were prepared in the same year using the same methodology, any time-related and technical batch effects cancel out throughout these comparisons.
