# Supplementary material for: Comparative profiling of surgically resected primary tumors and their lymph node metastases in small-cell lung cancer
Source: ESMO Open. 2025 Mar 18;10(4):104514. doi: 10.1016/j.esmoop.2025.104514 (PMC11964634; doi:10.1016/j.esmoop.2025.104514)
Supplement: Supplementary Data [file mmc3.docx]

**Supplementary Figure 1. Principal component analysis (PCA) and t-distributed stochastic neighbor embedding (t-SNE) of non-batch-corrected RNAseq data, according to technical parameters.** (*A*) sample type; (*B*) total area of the tissue core; (*C*) used lysis buffer; (*D*) type of surgery; (*E*) adjuvant chemotherapy; (*F*) year of surgery. Light grey color indicates missing metadata for the given samples.

**Supplementary Figure 2. Principal component analysis (PCA) of raw, non-batch-corrected RNAseq data and the data corrected for different batch effects in the case of samples with complete metadata.** (***A***) total area of the tissue core; (***B***) used lysis buffer; (***C***) type of surgery; (***D***) adjuvant chemotherapy; (***E***) year of surgery for genes of interest (CD47, c-myc, l-myc, DLL3, Ezh2, LSD1, mTOR, PD-L1, PIK3, and YAP1).

**Supplementary Figure 3. Volcano plot of gene expression differences between primary tumors and lymph node (LN) metastases.** Each dot represents a single gene. Non-significantly differentially expressed genes are represented with grey dots. Genes of interest are labeled with black. Genes with significant differential expression after correction for multiple testing are labeled with blue (down-regulated in LNs) or red (up-regulated in LNs).

**Supplementary Figure 4. Representative immunohistochemistry (IHC) images of each investigated marker in primary tumors and corresponding lymph node metastases.** Paired tissue samples originate from the same SCLC patient. The representative images were captured with a 20x objective lens. The positive cells were visualized with 3-3′ -diaminobenzidine (DAB), and the nuclei were labeled with hematoxylin. The top left row corresponds to the routine H&E-staining.

**Supplementary Figure 5. Hierarchical clustering (*A*) and principal component (PC) analysis (*B*) of primary (P) tumors and lymph node (LN) metastases based on RNA expression of molecules of interest.** Using the RNA expression data of YAP1, CD47, c-myc, DLL3, Ezh2, l-myc, LSD1, mTOR, PD-L1 and PIK3 samples could not be classified according to their site of origin.

**Supplementary Figure 6. Molecular subtypes of SCLC primary tumors and their corresponding lymph node (LN) metastases.** Blue cells indicate no subtype change between the two samples of different origins (y-axis: primary tumor, x-axis: LN metastasis), while red marks a change in the molecular subtype. The number of patients affected and not affected by subtype change is shown in the corresponding cells. The most common changes include SCLC-A to either SCLC-AN or SCLC-QN, and SCLC-QN to either SCLC-AN or SCLC-N. Interestingly, SCLC-AN primary tumors tended to have the same subtype in their LN metastasis. None of the LN metastases had SCLC-P subtype, despite two SCLC-P primary tumors.
